# Developing a phantom for simulating robotic-assisted complete mesocolic excision using 3D printing and medical imaging

**DOI:** 10.1186/s12893-024-02353-y

**Published:** 2024-02-26

**Authors:** Peter Hertz, Claus Anders Bertelsen, Kim Houlind, Lars Bundgaard, Lars Konge, Flemming Bjerrum, Morten Bo Søndergaard Svendsen

**Affiliations:** 1 Department of Surgery, Hospital Lillebaelt, University of Southern Denmark, Sygehusvej 24, Kolding, 6000 Denmark; 2 Department of Regional Health Research, University of Southern Denmark, Odense, Denmark; 3 Copenhagen Academy for Medical Education and Simulation (CAMES), Center for HR and Education, The Capital Region of Denmark, Copenhagen, Denmark; 4 Department of Surgery, Copenhagen University Hospital – North Zealand, Hillerød, Denmark; 5 Department of Vascular Surgery, Hospital Lillebaelt, University of Southern Denmark, Kolding, Denmark; 6 Department of Surgery, Hospital Lillebaelt Vejle, Colorectal Cancer Center South, University of Southern Denmark, Odense, Denmark; 7 Gastrounit, Surgical section, Copenhagen University Hospital - Amager and Hvidovre, Hvidovre, Denmark; 8 Department of Clinical Medicine, Faculty of Health and Medical Sciences, University of Copenhagen, Copenhagen, Denmark; 9 Department of Computer Science, Faculty of Science, University of Copenhagen, Copenhagen, Denmark

**Keywords:** Education, Simulation, Training, Robotic surgery, Complete mesocolic excision, Simulator, Development, 3D Printing, Competency, Assessment

## Abstract

**Background:**

Robotic-assisted complete mesocolic excision is an advanced procedure mainly because of the great variability in anatomy. Phantoms can be used for simulation-based training and assessment of competency when learning new surgical procedures. However, no phantoms for robotic complete mesocolic excision have previously been described. This study aimed to develop an anatomically true-to-life phantom, which can be used for training with a robotic system situated in the clinical setting and can be used for the assessment of surgical competency.

**Methods:**

Established pathology and surgical assessment tools for complete mesocolic excision and specimens were used for the phantom development. Each assessment item was translated into an engineering development task and evaluated for relevance. Anatomical realism was obtained by extracting relevant organs from preoperative patient scans and 3D printing casting moulds for each organ. Each element of the phantom was evaluated by two experienced complete mesocolic excision surgeons without influencing each other’s answers and their feedback was used in an iterative process of prototype development and testing.

**Results:**

It was possible to integrate 35 out of 48 procedure-specific items from the surgical assessment tool and all elements from the pathological evaluation tool. By adding fluorophores to the mesocolic tissue, we developed an easy way to assess the integrity of the mesocolon using ultraviolet light. The phantom was built using silicone, is easy to store, and can be used in robotic systems designated for patient procedures as it does not contain animal-derived parts.

**Conclusions:**

The newly developed phantom could be used for training and competency assessment for robotic-assisted complete mesocolic excision surgery in a simulated setting.

**Supplementary Information:**

The online version contains supplementary material available at 10.1186/s12893-024-02353-y.

## Background

More than 150,000 patients were diagnosed with colorectal cancer in the United States in 2022 [1] and 520,000 in Europe in 2020 [2]. Surgery remains the cornerstone in the treatment of colorectal cancer [1].

Many institutions use complete mesocolic excision (CME) in the surgical treatment of colon cancer, either as an open, laparoscopic, or robot-assisted procedure. The number of robot-assisted procedures has increased in recent years [3–5] The surgical principles of CME are dissecting in the embryological mesocolic plane envelope, central vascular ligation to obtain excision of central lymph nodes, and division of the bowel in an adequate distance from the tumour, i.e. 10 cm or more to the tumour [6]. Some surgeons are still opposed to CME due to the potential risk of critical intraoperative complications [7–10]. This is despite the safety of right-sided CME being shown in a randomized trial [10] and a long-term causal treatment effect with a reduction of both the risk of recurrence and the overall survival after right-sided CME found in a population-based cohort study [11].

There is a general perception that right-sided CME is a technically challenging and demanding procedure due to the wide exposure of the superior mesenteric vein and the anatomy relating to the pancreas. Hence, developing sufficient training modalities that allow surgeons to develop the necessary competencies is paramount for high-quality procedure training. Tejedor et al. recently reported international consensus on CME techniques and curricula content, including recommendations on anatomy teaching, hands-on training courses with a cadaver and proficiency-based assessments, including pathological outcomes [12]. The introduction of a standardised CME training setup has resulted in superior pathology specimens [13] why the pathological assessment of the removed specimen should play a role in competency assessment.

It can be challenging to acquire and ensure robot-assisted CME competencies, as access to cadaver training is a limiting factor and animal models are not optimal due to differences in anatomy.

However, developing training phantoms in materials compliant with operating theatre standards will make robotic training easily accessible to surgeons. Simulation phantoms developed with the aid of 3D printing technologies are emerging and it is now possible to train and assess surgical competencies for specific robotic procedures on these phantoms [14]. Other phantoms have demonstrated the possibility of pathological assessment integrated into the competency assessment [15]. However, no phantom has previously been developed for robotic right-sided CME hemicolectomies.

The aim of this study was to build a surgical phantom with the following specifications: providing trainees with an opportunity to learn the correct anatomy, allowing a pathological assessment of the removed specimen and making it possible to assess the competency level of trainees. Furthermore, it should be easy to store and made of non-organic material, making it possible to use in a surgical robotic system that is also used for patients.

We describe the development of an inanimate phantom, using 3D printing based on actual patient computerized tomography (CT) scans, resembling human tissue, thus making training and competency assessment of robotic-assisted right-sided CME hemicolectomies possible.

## Methods

### Reporting of findings

The developer group included the principal investigator (PH), an engineer experienced with 3D printing (MS), medical education researchers and surgeons (LK, KH, and FB), and experienced CME surgeons (CAB and LB).

We will report our work in four phases inspired by the Educational Design Framework (EDF) described by Ahmed Ghazi [16]. The framework is designed to guide the process when building a complex simulation phantom for procedure simulation with the highest possible educational impact. The EDF helps to identify and define relevant and irrelevant elements through four phases, where each phase builds on the results from the previous .

The EDF starts in phase #1 by gathering the physicians’ requirements for the phantom before translating these into engineering tasks in phase #2 using the criteria: anatomic realism, procedural relevance, physiological realism, and methods for competency assessment.

Phase #3 is about establishing a consensus on the overall utility and relevant detailed specifications. Based on these, a prototype is built and tested in phase #4.

## Results

### The first phase

#### Phantom development driven by the need for competency assessment

Building a phantom based on the content of an assessment tool ensures future competency assessment when performing procedures on the phantom.

When assessing competencies in a simulated setting on a phantom or in the clinical setting on actual patients, the results should be reported together with data on validity evidence [17]. The complete mesocolic excision competency assessment tool (CMECAT) is a technical assessment tool with a procedure-specific checklist for laparoscopic CME [18]. Validity evidence has previously been described for CMECAT. In addition, Benz et al. have proposed a grading scale for the pathological assessment of removed CME specimens [19]. The items relevant to competency assessment [18] and the items relevant for performing a pathological assessment [19] were used as the content in the first phase of the EDF framework [16].

### The second phase

#### Translation of the content to engineering tasks

The CMECAT [18] has 48 procedure-specific items for right-sided CME surgery. The Benz classification [19] relies on the morphology of the specimen, including the estimated level of division of tumour supplying arteries and assessment of the plane of dissection.

#### 3D reconstruction

**Fig. 1:**
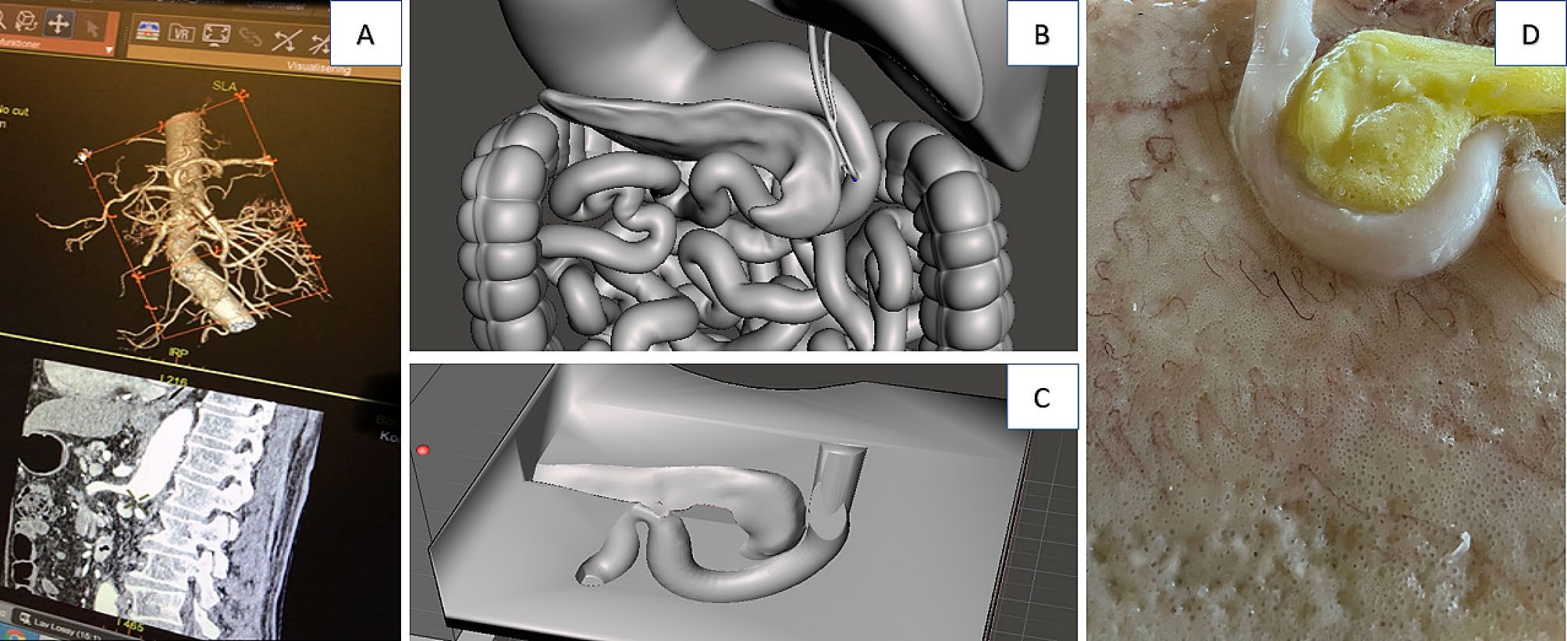
(**A**) Isolation and the extraction of the superior mesenteric artery. (**B**) Posterior view of the computer-aided design of the gastrointestinal tract and the pancreas. (**C**) Digital image of the casting form for the pancreas. (**D**) The final retroperitoneum surface with the duodenum and pancreas (yellow)

Every subtask of the CMECAT concerning anatomy was analyzed and the vascular anatomy and relations were solved by 3D reconstruction from preoperative CT imaging. We used both the ITK-SNAP [20] and the AW-server [21] software applications to extract relevant organ systems and their relations from Digital Imaging and Communications in Medicine (DICOM) files of contrast CT scans. Figure 1a shows the isolation and extraction of the superior mesenteric artery from a CT scan using AW-server [21]. The bowel, retroperitoneum, and pancreas were created by computer-aided design (Fig. 1b–d).

#### 3D printing

One limitation of 3D printing is the biomimicking properties of the available printing materials [22]. We used a known method to produce simulation phantoms by 3D printing, casting moulds in hard materials and making the phantoms of another moldable material [23].

If the organs extracted from the CT scan were improper for casting reproduction, e.g. two vessels had multiple connections or had unsuitable morphology, it was edited by using Meshmixer [24] software application before printing. The correct anatomical morphology was recreated after the casting production (Fig. 1a–c). The casting moulds were printed on Prusa printers, MK3, (Prusa Research, Prague, Czech Republic).

#### Phantom materials

The individual parts of the phantom were made by injecting two-component silicone into the casting forms. The silicone used (Smooth-On, Inc. 5600 Lower Macungie Road, Macungie, Pennsylvania, USA) could be altered using additives (Slacker, Smooth-On, Inc. 5600 Lower Macungie Road, Macungie, Pennsylvania, USA) to the desired properties regarding density and tear strength resembling the human tissue properties. After production, the organ systems were reconnected, and anatomical relations were reestablished to match the index CT scan. The anatomical structures were coated with materials alternating the adherence capability of the silicone, enabling different levels of adherence to related organs or structures, thereby mimicking the foetal planes relevant in surgery.

### The third phase

#### Consensus

To reach an expert consensus on the overall utility and anatomical components of the phantom, CAB, LB, and their colleagues supplied information on which anatomical structures are essential to include to achieve a realistic surgical dissection and assessment of CME on the phantom. Tissue realism was inspired by real procedure videos and pathological specimen pictures. The opinions of the experts were collected without the possibility of the experts influencing each other’s answers.

### The fourth phase

#### Testing and feedback

**Fig. 2:**
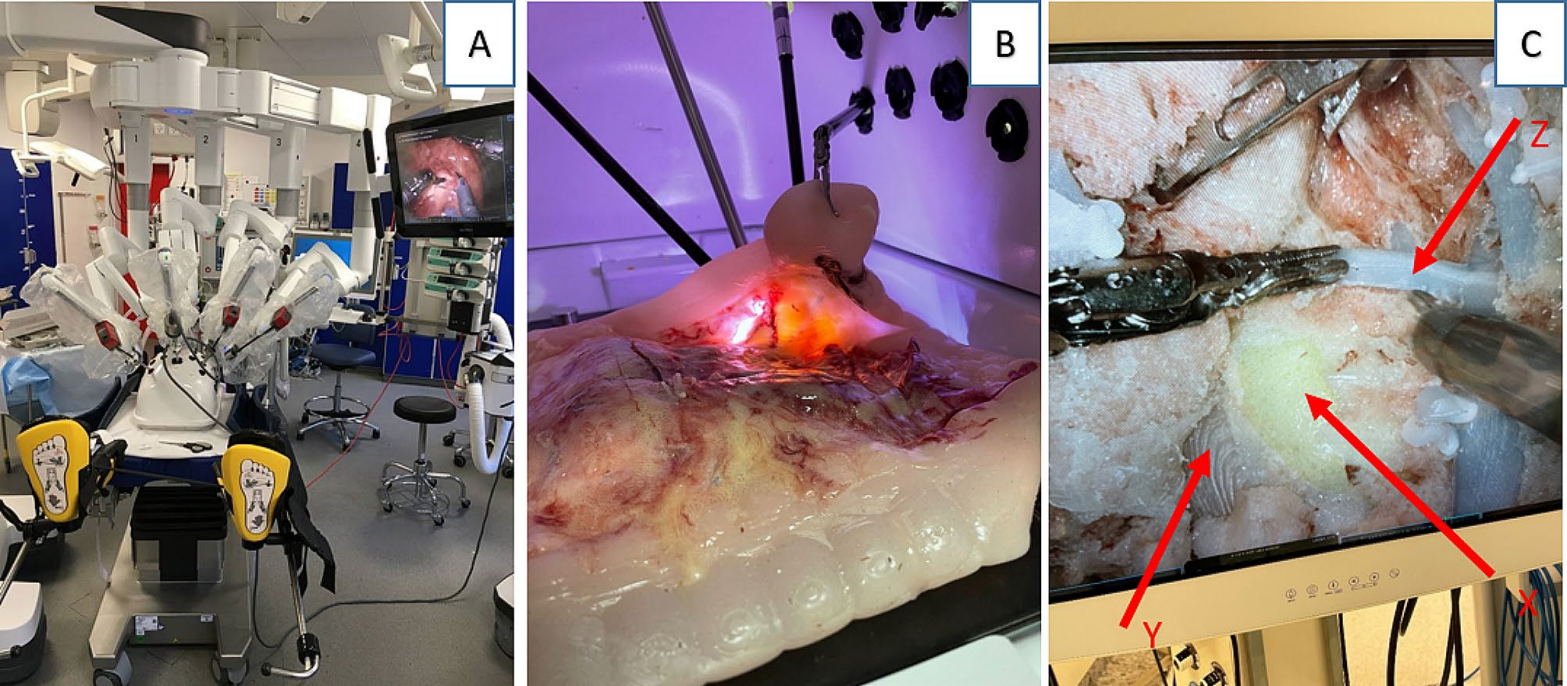
(**A**) Set up and test in a robotic operation theatre. (**B**) The interior of the abdominal training box. The caecum and the mesentery of the terminal ileum have been mobilized from the retroperitoneal fascia and lifted. (**C**) Vascular anatomy anteriorly to the exposed pancreatic head (X: Pancreas. Y: Duodenum. Z: Gastro colic trunk of Henle)

**Fig. 3:**
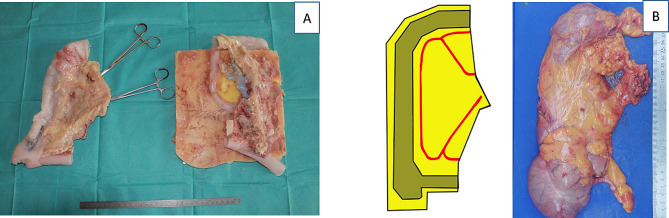
(**A**) Photography of the resected specimen and the remaining model (retroperitoneal fascia, duodenum and head of the pancreas. (**B**) Schematic drawing and photography of the perfect human specimen recreation based on the German classification of pathological specimens after right hemicolectomy proposed by Benz et al. [19]

Prototype testing was done by creating sub-task models, e.g. a plane of dissection model, vascular transection model, bowel stapling model etc. Each of the subtask models was tested and altered in iterations until a consensus was reached between the two experienced CME surgeons, LB and CAB. Feedback was given on the functional and physical resemblance of the models. After the sub-task models were approved, a complete phantom was assembled and tested in the robotic system (Fig. 2a–c). The resected specimens were compared with ones obtained from actual operations to evaluate the possibility of pathological assessment of the specimens (Fig. 3a and b).

### Competency assessment

We were able to build an inanimate phantom where 35 of 48 items on the CMECAT assessment tool can be assessed. The items that could not be integrated into the phantom were six items concerning bleeding, patient positioning, use of an assistant, thermal injuries, and four items relating to the use of retracting tools. Supplementary 1 is the original assessment tool by Haug et al. [18]. (The items that could not be integrated into the model are 1,2,4,5,8,11,17,22,28,33,37,40,46)

**Fig. 4:**
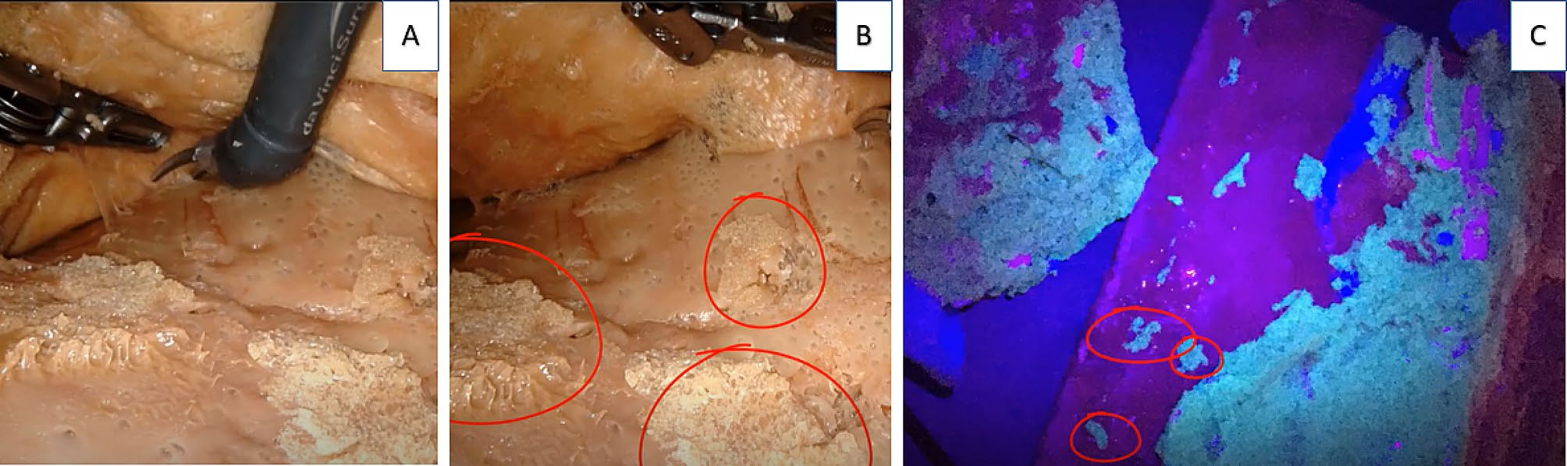
(**A**) Dissection plane between the mesocolon and the retroperitoneal fascia, robotic view. (**B**) Plane of dissection with markings on unresected mesocolic tissue on the retroperitoneal fascia. (**C**) The phantom is exposed to ultraviolet light, including the marking of unresected mesocolic tissue

It is possible to assess the removed specimen using the Benz classification [19]. To evaluate the plane of dissection, we added fluorophores to the mesocolic tissue enabling a postprocedure assessment with ultraviolet light as shown in Figs. 3b and 4a–c.

### Quantification of fluorescent signal

Fluorescence can be acquired by a standard camera by using an ultraviolet flashlight for the excitation of the fluorophores.

#### Quantification

**Fig. 5:**
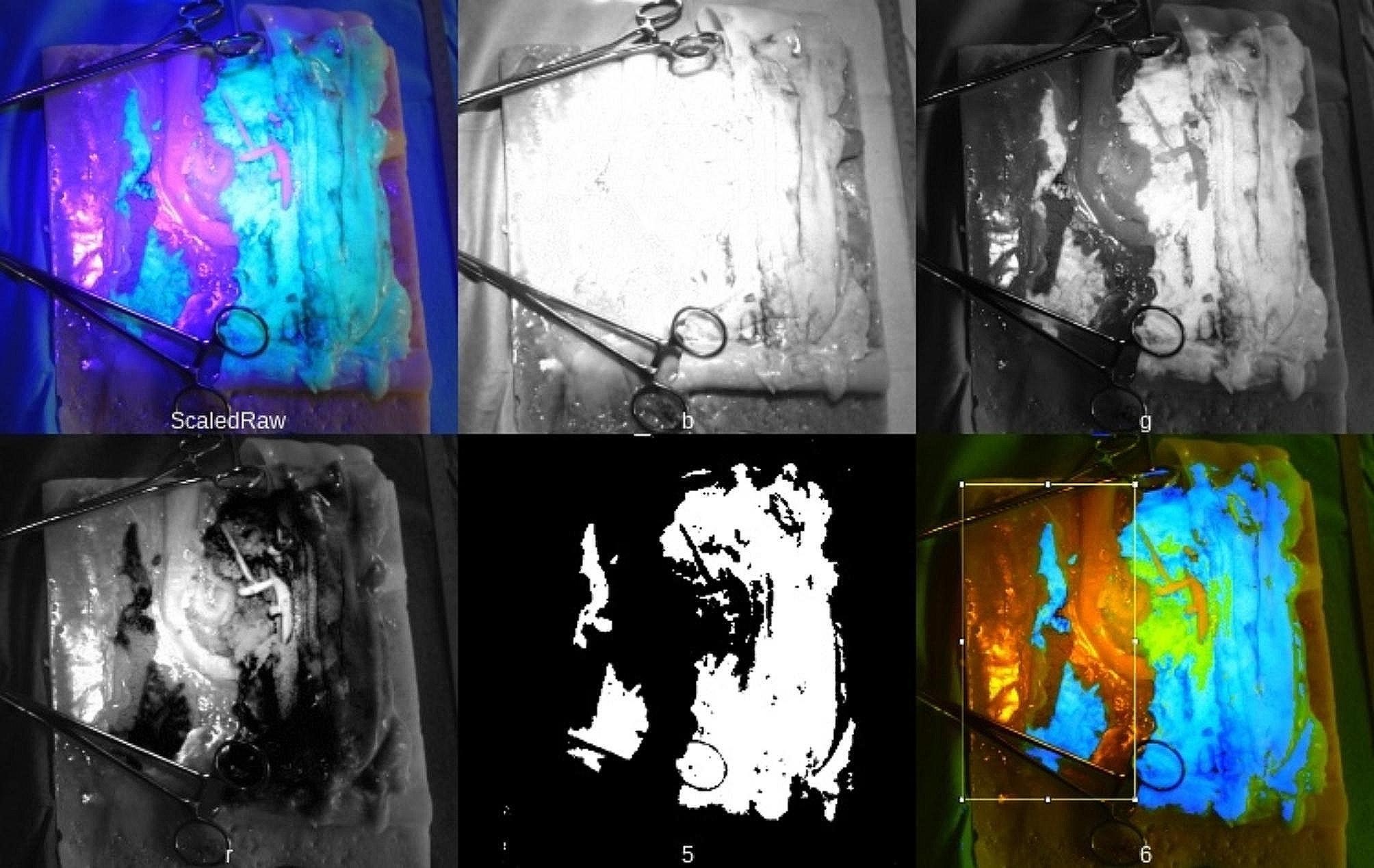
Photographs of the quantification of fluorescent signal for assessment of the plane of dissection. Upper row: raw image, blue, green. lower row: red, mask, false colour visualizing fluorescence signal

The image, as acquired by the camera, was split into red, green, and blue components. The blue channel contains the ultraviolet spillover into the blue colours and represents the excitation signal. We utilized the green channel as the provider of the signal, with red as the reference. A value of 1 was added to each pixel to avoid division errors and the resulting equation for the fluorescent image signal (FIM) is FIM = (*G* + 1)/(*R* + 1). *G* represents image pixels in the green channel and *R* in the red channel. The FIM was thresholded at an intensity of 50 and the resulting mask used for generating a false colour image highlighting residual mesocolic tissue on the retroperitoneal peritoneum. A region of interest is placed using ImageJ [25], providing a score of the percentage of residual tissue in the highlighted region of 11.76% in the test model. Image analysis of the plane of dissection was completed in Python 3.8 [26], using OpenCV [27] and Numpy libraries [28]. The image montage and measurement were completed in ImageJ [25]. The process is visualized in Fig. 5.

## Discussion

We designed an inanimate phantom using 3D printing for robotic right-sided CME with specifications based on earlier studies. We aimed to identify the relevant procedural steps for assessing surgical competency and specimen quality [18, 19]. The phantom developed is easy to store and does not contain animal-derived materials, making it applicable to use with robotic systems used on patients in the clinical setting.

### Methodological considerations

#### Anatomy

CME is considered complex mainly because the vascular structures of the right colon have a high degree of variability [29, 30]. Efforts have been made to standardize the procedure of central dissection [31–33], and standardized patient-specific preoperative planning using 3D models to improve anatomical understanding [34, 35].

Luzon et al. compared the vessel anatomy reconstructed as virtual 3D or 3D printed organs with the intraoperative measurements and found the correlations to be acceptable [36]. Creating models with anatomical accuracy and thus achieving better anatomical understanding might improve safety [37]. We have created a phantom that combines technical training with the advantage of learning the anatomical relations of important structures. It is possible to set up a simulation-based curriculum, including a preoperative planning step [35] and afterwards perform a robotic right-sided CME procedure on the expected anatomy. A perspective of the methodology used to create a phantom is the possibility of creating phantoms suitable for patient-specific rehearsals [38].

#### Phantom material

The phantom was built using platinum-cure silicone, which is easily stored, resulting in a phantom made of non-reactive and nonbiohazardous materials enabling use in actual operating theatres. However, one limitation in the usage of silicone is the inability to use electrocautery instruments. Knowledge of the principles of electrocautery or energy devices is rated essential as a prerequisite before basic robotic surgery [39]. Using electrocautery on the phantom was not considered crucial for the phantom as trainees mastered this before commencing robotic surgery. Building a phantom for robot-assisted surgery is easier than for conventional laparoscopic surgery due to the absence of haptic feedback in the robotic console. Therefore, the model only needs to have some degree of physical resemblance, mainly visual, but a high degree of functional task alignment [40].

#### Assessment of competency and training

We reported the development process using the EDF framework [16], which also describes the competency assessment using phantoms combined with either relevant metrics or expert rater assessment. We suggest the addition of a fifth phase to the framework called assessment of performance, i.e. evaluating the effect. This fifth phase should evaluate performance data on the phantom, thus ensuring the evaluation of the intended aim.

Using established assessment methods in the development of the phantom eases the possibility of measuring the competencies of the trainee. The performance data can also be used to evaluate the validity of automatic assessments such as the one we developed for the plane of dissection. We found that 11% of the mesocolic tissue was left behind in our test, and having performance data would make it possible to establish a training benchmark.

### Other ways to simulate surgery

Virtual reality (VR) simulation is a valuable adjunct to surgical training [41, 42]. To our knowledge, there are no VR simulators available for robotic CME training. A physical phantom distinguishes itself from VR modalities as it is not dependent on one specific robotic system, thus it can be used with any robotic surgical system available on the market. Another strength of using a phantom instead of a VR simulator is the possibility of applying the same pathology assessment to the simulated specimen as on the specimen retrieved from operations.

The use of cadavers or animal tissues is often considered the gold standard when training in advanced robotic-assisted surgical procedures. However, the technologies have now evolved enough to offer a more affordable alternative with easier access than these traditional training forms [14].

Future research should focus on the generalizability of surgical performance and assessment on the phantom by evaluation of performance data. The learning potential of the phantom in the laparoscopic setting could also be an area of future interest.

In conclusion, our phantom provides a new possibility to train and assess the advanced surgical procedure of robotic-assisted complete mesocolic excision. We have integrated anatomy and relevant procedural steps in a phantom that can be used with different robotic systems used in the clinical setting. Before implementing this phantom in curricula, performance data are needed to explore sources of validity.

### Electronic supplementary material

Below is the link to the electronic supplementary material.

Supplementary Material 1

## Data Availability

The datasets used and/or analysed during the current study are available from the corresponding author upon reasonable request.
