# Supplementary material for: Developing a phantom for simulating robotic-assisted complete mesocolic excision using 3D printing and medical imaging
Source: BMC Surg. 2024 Feb 26;24:72. doi: 10.1186/s12893-024-02353-y (PMC10897992; doi:10.1186/s12893-024-02353-y)
Supplement: Supplementary file 1 — Supplementary Material 1 [file 12893_2024_2353_MOESM1_ESM.pdf]

CMECAT

Laparoscopic Complete Mesocolic Excision Competency Assessment Tool

R

Name:	Assessor:	Video:

EXPOSURE

Please mark the answer box which best describes your response to the following statements.

	①	②	③	④	
1) Patients positioning to support exposure	INADEQUATE	SUBOPTIMAL	ADEQUATE	OPTIMAL	NOT APPLICABLE
2) Positioning of small bowel is performed	HARMFULLY	SUBOPTIMAL	COMPETENT	OPTIMAL	NOT APPLICABLE
3) Tissue dissection technique of the planes around pancreas and duodenum	DANGEROUS	SUBOPTIMAL	COMPETENT	OPTIMAL	NOT APPLICABLE
4) Use of the assistant	INADEQUATE	SUBOPTIMAL	ADEQUATE	OPTIMAL	NOT APPLICABLE
5) Were there any risks of thermal injuries?	DEFINITELY	POTENTIALLY	UNLIKELY	NEVER	NOT APPLICABLE
6) Key structures and landmarks were demonstrated	INADEQUATE	SUBOPTIMAL	ADEQUATE	OPTIMAL	NOT APPLICABLE
7) During the exposure of the operating field the bowels/tissue were	INJURED	POTENTIALLY DAMAGED	SAFELY HANDLED	PROTECTED THROUGHOUT	NOT APPLICABLE
8) If bleeding occurred, it was controlled	INADEQUATE	SUBOPTIMAL	ADEQUATE	OPTIMAL	NOT APPLICABLE
9) Damage to the mesentery during exposure can be described as	ONCOLOGICAL SIGNIFICANT	CONCERNING	MINIMAL	NO DAMAGE OBSERVED	NOT APPLICABLE
Score:					/

ILEOCOLIC VESSELS

Please mark the answer box which best describes your response to the following statements.

	①	②	③	④	
10) Use of dissection instruments to expose the ileocolic vessels	DANGEROUS	SUBOPTIMAL	COMPETENT	OPTIMAL	NOT APPLICABLE
11) Use of retracting tools (A-frame, swab, atraumatic retractor) to retract the colon and mesentery	DANGEROUS	SUBOPTIMAL	COMPETENT	OPTIMAL	NOT APPLICABLE
12) Tissue dissection technique of the planes around the ileocolic pedicles and superior mesenteric artery and vein	DANGEROUS	SUBOPTIMAL	COMPETENT	OPTIMAL	NOT APPLICABLE
13) The perivascular 360° exposure of the ileocolic vein	INADEQUATE	SUBOPTIMAL	ADEQUATE	OPTIMAL	NOT APPLICABLE
14) The perivascular 360° exposure of the ileocolic artery	INADEQUATE	SUBOPTIMAL	ADEQUATE	OPTIMAL	NOT APPLICABLE
15) The protection and exposure of landmarks before transection of the ileocolic vessels such as superior mesenteric vessels and pancreas	INADEQUATE	SUBOPTIMAL	ADEQUATE	OPTIMAL	NOT APPLICABLE
16) Unintended tissue damage and bleeding during perivascular dissection of the superior mesenteric vein	SIGNIFICANT	CONCERNING	MINIMAL	NO DAMAGE OBSERVED	NOT APPLICABLE
17) If bleeding occurred, it was controlled	INADEQUATE	SUBOPTIMAL	ADEQUATE	OPTIMAL	NOT APPLICABLE
18) The level of ileocolic vein transection in relation to the lateral border of superior mesenteric vein	SIGNIFICANTLY OFF	SUBOPTIMAL	ADEQUATE	OPTIMAL	NOT APPLICABLE
19) The level of ileocolic artery transection in relation to its origin	SIGNIFICANTLY OFF	SUBOPTIMAL	ADEQUATE	OPTIMAL	NOT APPLICABLE
20) Lymphadenectomy along the central aspects of the ileocolic pedicle	NOT PERFORMED	SUBOPTIMAL	ADEQUATE	OPTIMAL	NOT APPLICABLE
Score:					/

MIDDLE COLIC VESSELS

Please mark the answer box which best describes your response to the following statements.

	①	②	③	④	
21) Use of dissecting instruments to expose the middle colic vessels	DANGEROUS	SUBOPTIMAL	COMPETENT	OPTIMAL	NOT APPLICABLE
22) Use of retracting tools (A-frame, swab, atraumatic retractor) to retract the colon and mesentery	DANGEROUS	SUBOPTIMAL	COMPETENT	OPTIMAL	NOT APPLICABLE
23) Tissue dissection technique of the planes around middle colic pedicles and superior mesenteric artery and vein	DANGEROUS	SUBOPTIMAL	COMPETENT	OPTIMAL	NOT APPLICABLE
24) The perivascular 360° exposure of the MCV or the branch of the MCV	INADEQUATE	SUBOPTIMAL	ADEQUATE	OPTIMAL	NOT APPLICABLE
25) The perivascular 360° exposure of the MCA or the branch of the MCA	INADEQUATE	SUBOPTIMAL	ADEQUATE	OPTIMAL	NOT APPLICABLE
26) The protection and exposure of landmarks prior to transection of the middle colic vessels, such as the superior mesenteric vessels and pancreas	INADEQUATE	SUBOPTIMAL	ADEQUATE	OPTIMAL	NOT APPLICABLE
27) Unintended tissue damage and bleeding during perivascular dissection of the superior mesenteric vein	SIGNIFICANT	CONCERNING	MINIMAL	NOT DAMAGE OBSERVED	NOT APPLICABLE
28) If bleeding occurred, it was controlled	INADEQUATE	SUBOPTIMAL	ADEQUATE	OPTIMAL	NOT APPLICABLE
29) The level of the MCA transection in relation to its origin	SIGNIFICANTLY OFF	SUBOPTIMAL	ADEQUATE	OPTIMAL	NOT APPLICABLE

Colour description: Instrument handling | Tissue handling | Clinical safety | Oncological safety

CMECAT

Laparoscopic Complete Mesocolic Excision Competency Assessment Tool

30) The level of the of the MCV transection in relation to its origin	SIGNIFICANTLY OFF	SUBOPTIMAL	ADEQUATE	OPTIMAL	NOT APPLICABLE
31) Lymphadenectomy along the central aspects of the middle colic pedicle	NOT PERFORMED	SUBOPTIMAL	ADEQUATE	OPTIMAL	NOT APPLICABLE
Score:					/

GASTROCOLIC TRUNK (GCT)

Please mark the answer box which best describes your response to the following statements.

	①	②	③	④	
32) Use of dissecting instruments to expose GCT	DANGEROUS	SUBOPTIMAL	COMPETENT	OPTIMAL	NOT APPLICABLE
33) Use of retracting tools (A-frame, swab, atraumatic retractor) to retract the colon and mesentery	DANGEROUS	SUBOPTIMAL	COMPETENT	OPTIMAL	NOT APPLICABLE
34) Tissue dissection technique of the planes around GCT and superior mesenteric vein and artery	DANGEROUS	SUBOPTIMAL	COMPETENT	OPTIMAL	NOT APPLICABLE
35) The perivascular 360° exposure of the GCT related veins (right colic, superior right colic, gastroepiploic etc)	INADEQUATE	SUBOPTIMAL	ADEQUATE	OPTIMAL	NOT APPLICABLE
36) The protection and exposure of landmarks before transection of GCT related vessels, such as superior mesenteric vessels, pancreas, duodenum and stomach	INADEQUATE	SUBOPTIMAL	ADEQUATE	OPTIMAL	NOT APPLICABLE
37) If bleeding occurred, it was controlled	INADEQUATE	SUBOPTIMAL	ADEQUATE	OPTIMAL	NOT APPLICABLE
38) The level of the GCT related veins transection in relation to its origin	SIGNIFICANTLY OFF	SUBOPTIMAL	ADEQUATE	OPTIMAL	NOT APPLICABLE
Score:					/

MOBILISATION AND ANASTOMOSIS

Please mark the answer box which best describes your response to the following statements.

	①	②	③	④	
39) Use of dissecting tools during mobilisation	DANGEROUS	SUBOPTIMAL	COMPETENT	OPTIMAL	NOT APPLICABLE
40) Use of retracting tools (A-frame, swab, atraumatic retractor) when the mesentery and colon is retracted	DANGEROUS	SUBOPTIMAL	COMPETENT	OPTIMAL	NOT APPLICABLE
41) Use of stapling device	DANGEROUS	SUBOPTIMAL	COMPETENT	OPTIMAL	NOT APPLICABLE
42) The mobilisation of the right colon with safe division of the gastrocolic and lateral peritoneal attachments	DANGEROUS	SUBOPTIMAL	COMPETENT	OPTIMAL	NOT APPLICABLE
43) Transection of the terminal ileum proximal to the ileocecal valve	DANGEROUS	SUBOPTIMAL	COMPETENT	OPTIMAL	NOT APPLICABLE
44) The protection and exposure of landmarks before transection the distal colon, such as duodenum pars 1-3, pancreas, smooth retroperitoneal surface and Gerota's fascia	INADEQUATE	SUBOPTIMAL	ADEQUATE	OPTIMAL	NOT APPLICABLE
45) Transection of colon and anastomosis was performed	DANGEROUS	SUBOPTIMAL	COMPETENT	OPTIMAL	NOT APPLICABLE
46) If bleeding occurred, it was controlled	INADEQUATE	SUBOPTIMAL	ADEQUATE	OPTIMAL	NOT APPLICABLE
47) Damage to the mesocolic side of the specimen can be described as	ONCOLOGICAL SIGNIFICANT	CONCERNING	MINIMAL	NO DAMAGE OBSERVED	NOT APPLICABLE
48) Proximal and distal resection margin in relation to tumour	TOO CLOSE	SUBOPTIMAL	ADEQUATE	OPTIMAL	NOT APPLICABLE
Score:					/

TOTAL SCORE:	/

Feedback/Commentary:

Colour description: Instrument handling | Tissue handling | Clinical safety | Oncological safety
